# The essential oil of *Hyptis crenata* Pohl ex Benth. presents an antiedematogenic effect in mice

**DOI:** 10.1590/1414-431X20209422

**Published:** 2021-01-25

**Authors:** A.N. Coelho-de-Souza, R. Alves-Soares, H.D. Oliveira, Y.A. Gomes-Vasconcelos, P.J.C. Souza, T. Santos-Nascimento, K.A. Oliveira, L.R.L. Diniz, J. Guimarães-Pereira, J.H. Leal-Cardoso

**Affiliations:** 1 Laboratório de Fisiologia Experimental, Instituto Superior de Ciências Biomédicas, Universidade Estadual do Ceará, Campus do Itaperi, Fortaleza, CE, Brasil; 2 Programa de Pós-Graduação em Bioquímica, Universidade Federal do Ceará, Fortaleza, CE, Brasil; 3 Departamento de Farmácia, Universidade Federal do Pará, Belém, PA, Brasil; 4 Faculdade do Vale do Jaguaribe, Fortaleza, CE, Brasil; 5 Faculdade do Nordeste da Bahia, Salvador, BA, Brasil; 6 Laboratório de Eletrofisiologia, Instituto Superior de Ciências Biomédicas, Universidade Estadual do Ceará, Campus do Itaperi, Fortaleza, CE, Brasil

**Keywords:** Medicinal plants, Essential oil, Hyptis crenata, Antiedematogenic activity, Inflammation

## Abstract

*Hyptis crenata*, commonly known as “salva-do-Marajó”, “hortelã-do-campo”, and “hortelãzinha”, is used in folk medicine in Northeast Brazil as tea or infusion to treat inflammatory diseases. Due to the pharmacological efficacy and the low toxicity of the essential oil of *Hyptis crenata* (EOHc), we decided to investigate the EOHc antiedematogenic effect in experimental models of inflammation. EOHc was administrated orally at doses of 10-300 mg/kg to male Swiss albino mice. Paw edema was induced by subcutaneous injection in the right hind paw of inflammatory stimuli (carrageenan, dextran, histamine, serotonin, and bradykinin) 60 min after administration of EOHc. EOHc significantly inhibited the induced edema. The inhibitory effect of EOHc on dextran-induced edema extended throughout the experimental time. For the 30, 100, and 300 mg/kg doses of EOHc, the inhibition was of 40.28±1.70, 51.18±2.69, and 59.24±2.13%, respectively. The EOHc inhibitory effect on carrageenan-induced edema started at 10 mg/kg at the second hour (h) and was maintained throughout the observation period. At 30, 100, and 300 mg/kg doses the inhibition started earlier, from 30 min. At the edema peak of 180 min, 56, 76, and 82% inhibition was observed for 30, 100, and 300 mg/kg doses, respectively. Additionally, the effect of EOHc on carrageenan-induced paw edema was influenced by the time of administration. The EOHc also inhibited myeloperoxidase activity. In conclusion, the EOHc showed a potent effect, both preventing and reversing the edema, consistent with its anti-inflammatory use in folk medicine.

## Introduction

Plants of the *Hyptis* genus belong to the Lamiaceae family, which ranks third in ethnopharmacological importance (1,2). In Brazil, *Hyptis* plants occur in several states of the North, Northeast (3), Midwest, and Southeast regions, usually in the Amazon rainforest (4) and Cerrado (1) where they are used for different purposes, ranging from appetizer, food flavoring, and bath aromatization, to treatment of various diseases including inflammation (5,6), respiratory (7) and gastrointestinal disorders (3,6,8), constipation, and arthritis (7,9). In the Brazilian Northeast, *H. crenata* is used in folk medicine in the form of tea or infusion. *H. crenata*, commonly known as “salva-do-Marajó”, “hortelã-do-campo”, and “hortelãzinha”, is rich in essential oils (EO), which main constituents are camphor, 1.8-cineole, and alpha-pinene (10,11).

Some biological activities of *H. crenata*, such as antimicrobial (12), bactericidal, larvicide (13), antioxidant (5), gastroprotective (8), and hepatoprotective (14), are already documented. Recent studies from our research group have shown that the essential oil of *Hyptis crenata* (EOHc) has low acute toxicity by the oral route (10). It demonstrated a hepatoprotective effect in sepsis-induced liver dysfunction at 100 mg/kg (*po*) during 14 days (14). EOHc has also shown a gastroprotective action (8), which was attributed to α-pinene, one of its major constituents (3). The low acute toxicity of EOHc was demonstrated through the value of its median lethal dose (LD_50_) that was estimated to be greater than 2000 mg/kg (10).

The EOHc has demonstrated pharmacological efficacy and low toxicity, it is abundant in plant parts, and its use is common in folk medicine to treat swelling and inflammation of the limbs. Thus, we aimed to investigate the EOHc antiedematogenic effect.

## Material and Methods

### Essential oil extraction

**Figure 1:**
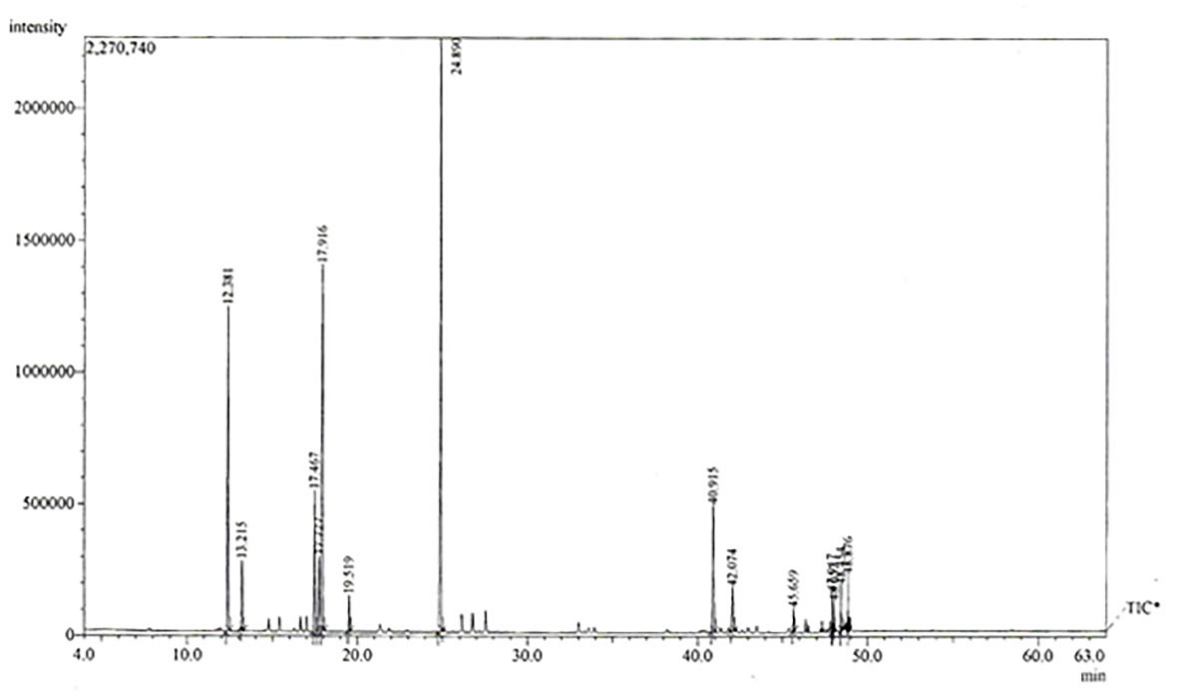
Gas chromatogram (GC-MS) of essential oil of leaves of *Hyptis crenata*. The peaks correspond to the retention time for the constituents identified.

The EOHc was obtained from leaves and branches of *H. crenata* by steam distillation (Detiller MA480, Marconi^®^, Brazil) as described for other essential oils (8,14–18). The plant was collected (January 2011) in the city of São Raimundo das Mangabeiras, Maranhão State, Brazil (7° 1′ 19- S, 45° 28′ 51- W). The identification was confirmed by Dr. Oriel Herera Bonilla (Ecology Laboratory, Brazil) and a voucher sample (No. 000106) was deposited at the Marlene Freitas da Silva herbarium (Brazil). Chemical constituents of the EOHc were kindly determined by Dr. Afrânio Aragão Craveiro from Technological Development Park of the Federal University of Ceará (PADETEC/UFC), by gas chromatography coupled to mass spectrometry (GC-MS, Hewlett-Packard 6971, USA). Analysis conditions were as follows: column of dimethylpolysiloxane DB-1 fused silica capillary column (30 m×0.25 mm; 0.1 μm); helium (1 mL/min) as carrier gas; injector temperature: 250°C; detector temperature: 200°C; column temperature: 35-180°C at 4°C/min and 180-250°C at 10°C/min; and mass spectra: electronic impact 70 eV. The compounds were identified (Figure 1 and Table 1) using mass spectral library search.

**Table 1:** Chemical composition of the essential oil of *Hyptis crenata* (EOHc), based on retention time.

Peak	Compound	Retention time	Area (%)
1	α-pinene	12.381	15.24^*^
2	Camphene	13.215	3.23
3	ρ-cymene	17.467	6.85
4	l-Limonene	17.727	3.94
5	1.8-cineole (Eucalyptol)	17.916	19.76^*^
6	γ-terpinene	19.519	1.73
7	Camphor	24.890	33.62^*^
8	β-caryophyllene	40.915	8.00^*^
9	Aromadendrene	42.074	2.95
10	Ledene	45.659	0.99
11	Caryophyllene oxide	47.917	0.84
12	Viridiflorol	48.052	0.85
13	10-epi-γ-eudesmole	48.454	0.96
14	Caryophyllene oxide	48.876	1.05
Total			100.00

Main compounds identified using mass spectral library search.

### Drugs and solutions

All salts and drugs used were of analytical purity. Carrageenan, dextran, indomethacin, histamine, serotonin, bradykinin, hexadecyltrimethylammonium bromide (CTAB), o-dianisidine dihydrochloride, hydrogen peroxide, and cyproheptadine were acquired from Sigma-Aldrich Chemical Corporation (USA). Tween 80 and NaCl were from Reagem (Brazil). Solutions were prepared by adding pure substance to sterile saline (0.9% NaCl). EOHc was prepared in sterile saline, containing Tween 80, 0.1% v/v, followed by automatic stirring. After homogenization, the solution was administered by the orogastric route.

### Animals

Male Swiss albino (*Mus musculus*) mice (30-40 g) were obtained from Christus University Center (UNICHRISTUS) vivarium and kept at the vivarium of the State University of Ceará (UECE) in a box of polypropylene, at a temperature of 23±2°C, 12 h dark/light cycle, and free access to water and food. The experimental protocol was approved by the Committee on Ethics in the Use of Animals of the State University of Ceará (CEUA/UECE; protocol number 2960651/2015) and followed the ethical principles on manipulation and use of laboratory animals of the Brazilian Society of Science in Laboratory Animals.

### Paw edema induction

Paw edema was induced by intra-plantar subcutaneous injection in the right hind paw of 50 μL of the inflammatory stimuli carrageenan or dextran (both at 1%), histamine (50 nmol/paw), serotonin (1 µg/paw), or bradykinin (3 nmol/paw), 60 min after intragastric administration of EOHc, indomethacin (10 mg/kg), cyproheptadine (5 or 10 mg/kg), or EOHc vehicle (Tween 80, 0.05% v/v). For blockade of carrageenan- or dextran-induced edema, the EOHc was administered at the doses of 10, 30, 100, or 300 mg/kg; for inhibition of response to other inflammatory stimuli histamine (HA), serotonin (5-HT) or bradykinin (BK), the essential oil was used at the dose of 100 and 300 mg/kg, which represents less than 15% of the LD_50_. The contralateral paw (control) received, subcutaneously, an equal volume of 0.9% NaCl (the vehicle of dextran or carrageenan). Paw volume variation was measured with a plethysmometer (Panlab, S.L.U., Digital Water Plethysmometer Le 7500, USA) before (zero time), at the 30th and 60th min, and afterwards, every 60 min up to 240 min (for dextran), or up to 300 min (for carrageenan). Edema was considered to be the difference in paw volume measured at different time periods and time zero. In this study, the time course of carrageenan-induced paw edema was considered to have three phases, as in another study (15 [Bibr B16] [Bibr B17]): a first phase, occurring in the first 60 min after drug administration, resulting of the presence of histamine and serotonin; a second phase, from (61-120 min), named osmotic phase, thought to involve the kinin system (19); and a third phase, from (121-180 min), named cellular phase, triggered by different mediators, including prostaglandins or a mix of prostaglandins and slow-reacting substances.

To determine whether the time of administration would influence the effect of EOHc on carrageenan-induced edema, in another experimental series, EOHc (100 mg/kg) or vehicle was administered 60 and 30 min before, at the time of induction, or 30 min after edema induction. The paw edema was evaluated at 30, 60, 120, 180, 240, 300, 1440 (24 h), and 2880 (48 h) min after edema induction.

### Myeloperoxidase (MPO) activity

For MPO activity, paw edema was induced by intra-plantar injection in the right hind paw of 50 μL of inflammatory stimuli, carrageenan at 1%, 60 min after intra-gastric administration of EOHc (100 mg/kg) or vehicle (Tween 80, 0.05% v/v). The contralateral paw (control) received an equal volume of 0.9% NaCl. At the inflammatory peak, 180 min after edema induction, the animals were sacrificed, sub-plantar tissue was removed, and immediately processed for analysis of MPO activity according to the method described by Rao et al. (20). The MPO assay reaction mixture consisted of the supernatant, 0.5% hexadecyltrimethylammonium bromide (CTAB), 0.68 mg/mL o-dianisidine dihydrochloride, and 0.003% hydrogen peroxide. The absorbance of this mixture was measured at 450 nm. One unit of MPO activity was defined as the quantity of enzyme degrading 1.0 μmol of hydrogen peroxide per min at 25°C, reported as MPO × 10^3^ U/mg tissue.

### Statistical analysis

Data are reported as means±SE. The graph and the statistical analysis were done with the software Sigmaplot^®^ (version 11.0, Systat Software, USA). Two-way analysis of variance (ANOVA) was used to compare the means followed by Bonferroni test, a multiple comparison method. For area under curve (AUC) and myeloperoxidase activity graphics, we used one-way ANOVA, followed by Bonferroni test. Results showing a probability of occurrence of the null hypothesis less than 5% (P<0.05) were considered statistically different.

## Results

### Main constituents of EOHc

Figure 1 shows the chromatogram for analysis of the EOHc by GC-MS. As can be seen, four main peaks were identified, which correspond to the major constituents camphor (33.62), 1.8-cineole also known as eucalyptol (19.76%), α-pinene (15.24%), and β-caryophyllene (8.00%), followed by 10 smaller peaks. By analyzing the retention time, 100% of the constituents were identified (Table 1).

### Inhibition of dextran-induced edema

**Figure 2:**
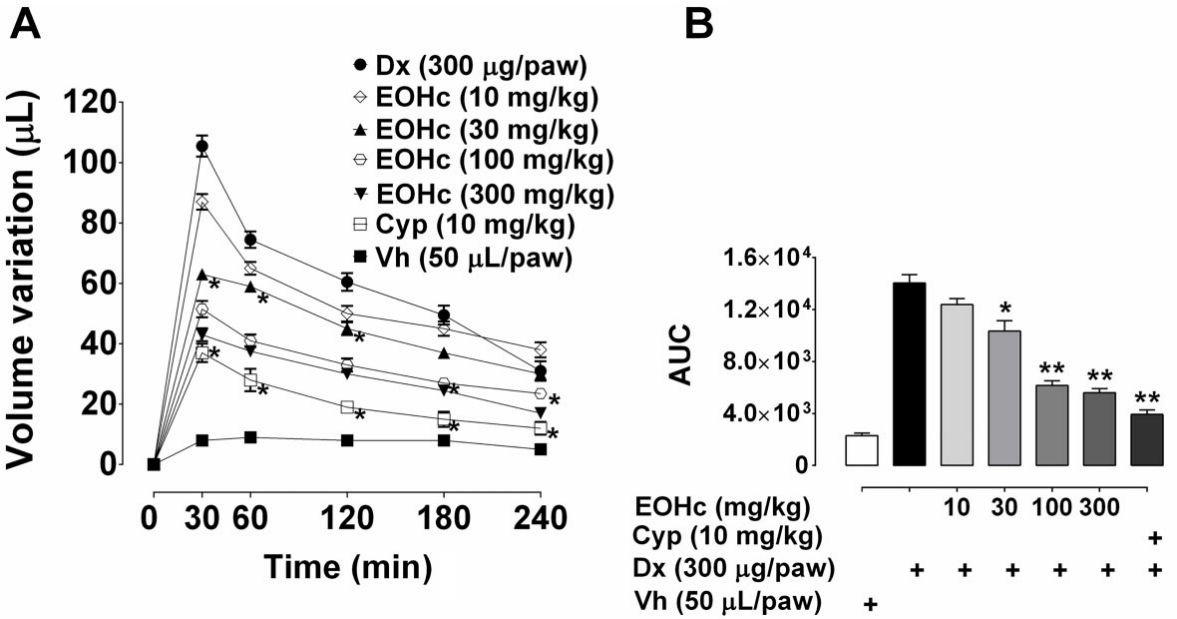
Effect of essential oil of *Hyptys crenata* (EOHc) on paw edema induced by dextran. **A** and **B,** Effect of several doses of EOHc (10-300 mg/kg, orally) and of cyproheptadine (Cyp, 10 mg/kg) on the time course (30-240 min) of edema induced by intraplantar injection of dextran (Dx, 300 µg/paw) or Vehicle (Vh, 0.9% NaCl solution, 50 μL/paw). Data are reported as means±SE (n=10). *P<0.05, **P<0.01 *vs* control (two-way ANOVA followed by Bonferroni's test).

EOHc significantly inhibited dextran-induced edema (Figure 2A and B). The inhibitory effect of EOHc extended throughout the experimental time (240 min) for doses ≥ 100 mg/kg. For the doses of 30, 100, and 300 mg/kg of EOHc, the observed inhibition corresponded to 40.28±1.70, 51.18±2.69, and 59.24±2.13%, respectively, of control (105.5±3.45 µL of paw volume variation) edema.

### Inhibition of carrageenan-induced edema

**Figure 3:**
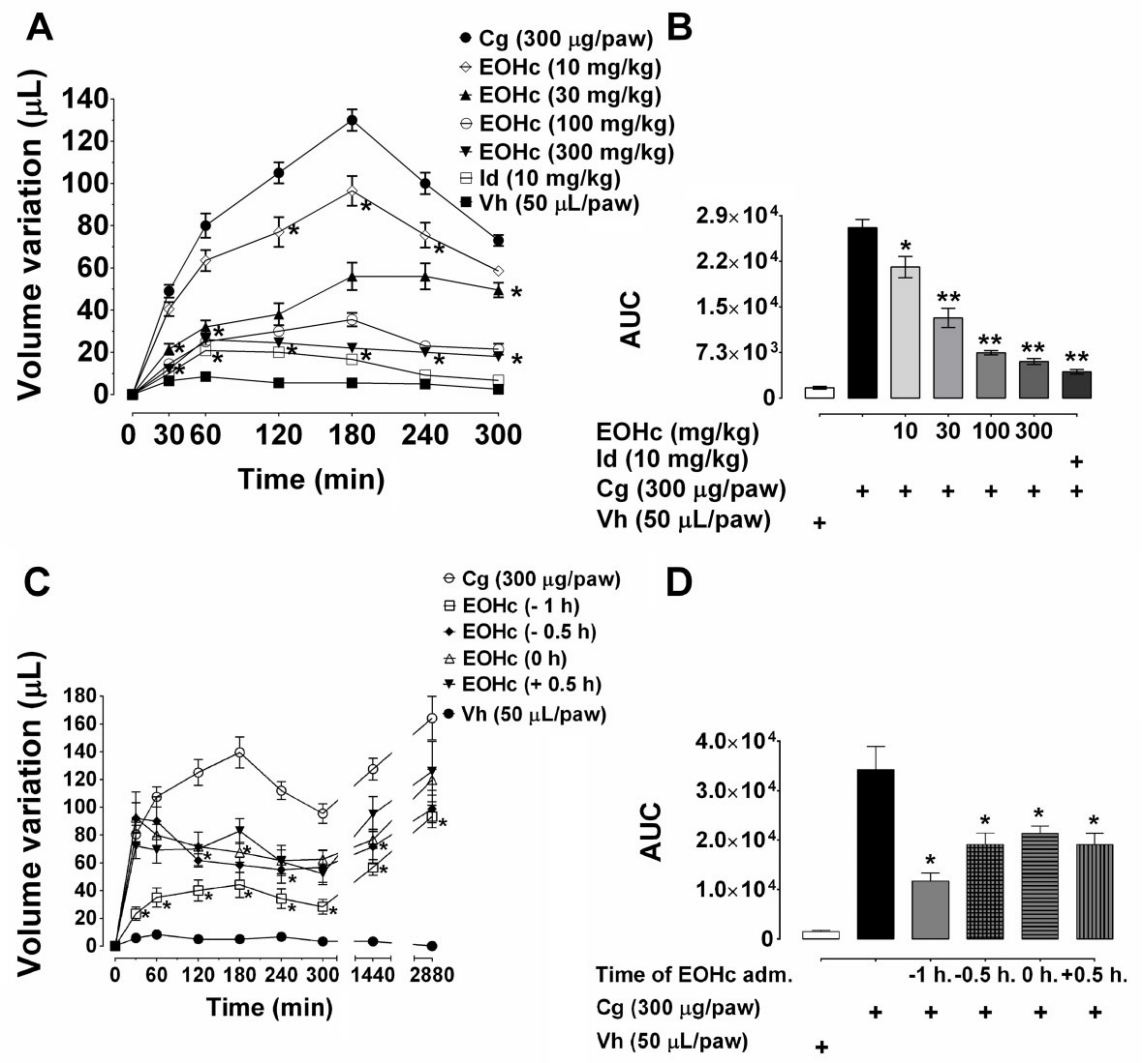
Effect of essential oil of *Hyptys crenata* (EOHc) on paw edema induced by carrageenan. **A** and **B**, Effect of several doses of EOHc (10-300 mg/kg, orally) and of indomethacin (Id, 10 mg/kg) on the time course (30-300 min) of edema induced by intraplantar injection of carrageenan (Cg, 300 µg/paw) or of its vehicle (Vh, 0.9% NaCl solution, 50 μL/paw). **C** and **D**, Influence of time of administration on the anti-inflammatory effect of EOHc (100 mg/kg) on the time course (30 min up to 48 h) of carrageenan-induced edema. The negative numbers (-1 and -0.5 h) indicate administration of EOHc before edema induction, the positive number (+0.5 h) indicates administration of EOHc after edema induction and 0 h indicates administration of EOHc concomitant with edema induction. *P<0.05, **P<0.01 *vs* control (two-way ANOVA followed by Bonferroni's test).

EOHc also had an inhibitory effect on edema induced by carrageenan (Figure 3A and B). For the dose of 10 mg/kg, this effect started from the 2nd h and was maintained throughout of the observation period. At the doses of 30, 100, and 300 mg/kg the inhibition started earlier, from 30 min. At the edema peak, 180 min, the paw volume inhibition corresponded to 56, 76, and 82% of the paw volume increase by carrageenan (130±5.1099 µL) for doses of 30, 100, and 300 mg/kg, respectively. Indomethacin (positive control) inhibited 84.5% of peak control edema.

### Influence of time of EOHc administration on carrageenan-induced paw edema

For this experimental series, the dose of 100 mg/kg of EOHc was used, because this was the lowest dose that had a similar effect to indomethacin, the positive control. In this way, any possible adverse effect would be minimized. The observation period was extended to 48 h after edema induction. Administered at different times relative to the time of administration of the edema inducing agent, the best EOHc effect occurred when EOHc was administered 60 min prior to the carrageenan (Figure 3D). Surprisingly, the effect of the EOHc lasted 48 h. Comparing the area under the curve for the inhibitory effect of EOHc (100 mg/kg), it can be seen that when administered 1 h before edema induction the inhibition corresponded to 65.71% of control (Figure 3C and D). For administration at 1 h before edema induction, the inhibitory effect was similar at all times of observation, 30, 60, 120, 240, 360, 1440 min (24 h), and 2880 min (48 h) (Figure 3C).

### Inhibition of paw edema induced by the autacoids histamine, serotonin, and bradykinin

**Figure 4:**
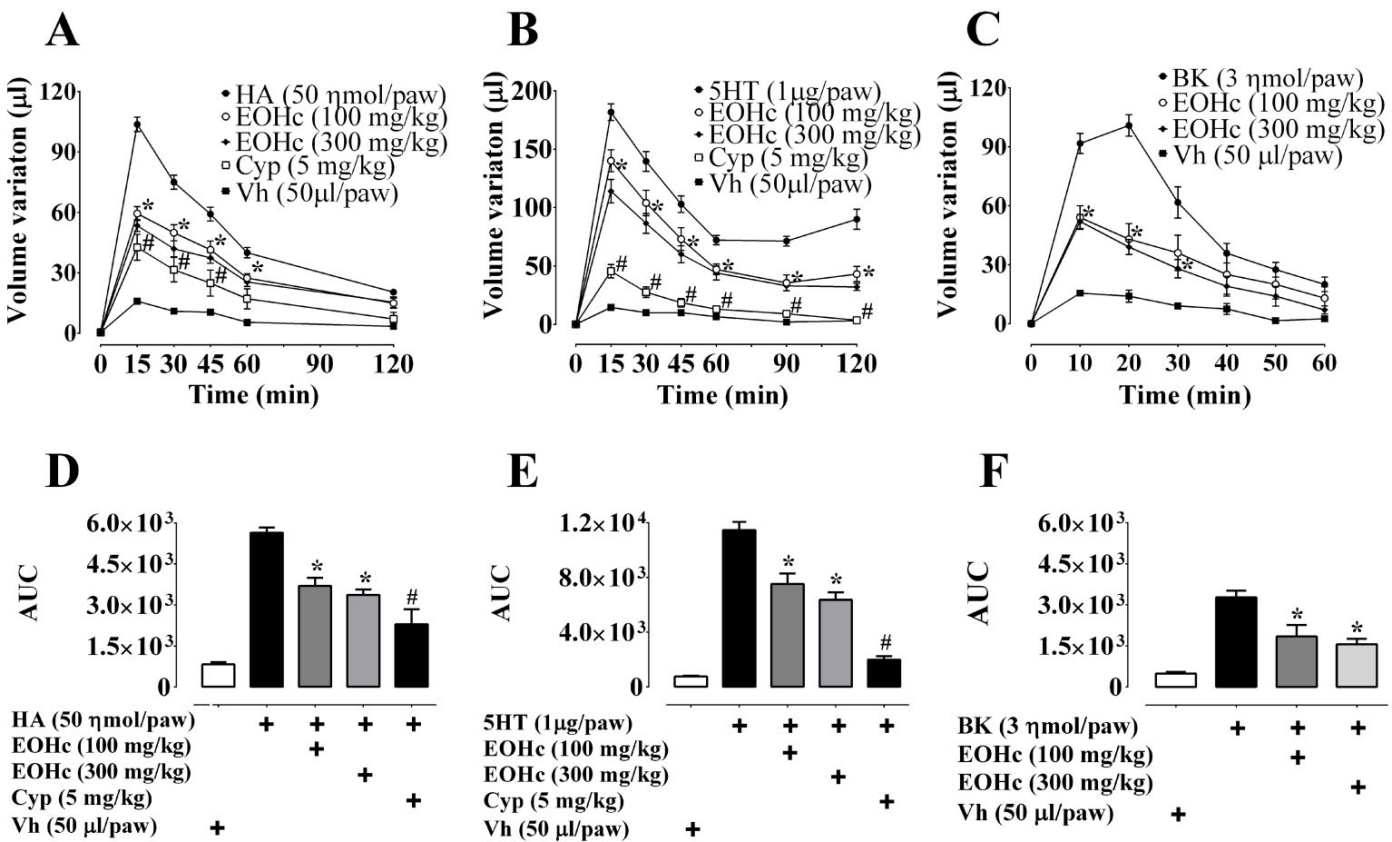
Effect of essential oil of *Hyptis crenata* (EOHc,100 and 300 mg/kg, orally) on paw edema induced by histamine, serotonin, or bradykinin. **A** and **D**, Effect of EOHc and of cyproheptadine (Cyp, 5 mg/kg) on the time course (15-120 min) of edema induced by intraplantar injection of histamine (HA, 50 ηmol/paw) or Vehicle (Vh, 0.9% NaCl solution, 50 μL/paw). **B** and **E**, Effect of EOHc and of Cyp (5 mg/kg) on the time course (15-120 min) of edema induced by intraplantar injection of serotonin (5HT, 1 μg/paw) or Vehicle (Vh, 0.9% NaCl solution, 50 μL/paw). **C** and **F**, Effect of EOHc on the time course (10-60 min) of edema induced by intraplantar injection of bradykinin (BK, 3 ηmol/paw) or Vehicle (Vh, 0.9% NaCl solution, 50 μL/paw). Data are reported as means±SE (n=10). *P<0.05 *vs* control, ^#^P<0.05 *vs* treated animals (EOHc, 100 and 300 mg/kg) (two-way ANOVA and one-way ANOVA followed by Bonferroni's test).

For this experimental series, the EOHc was used at the doses of 100 mg/kg and 300 mg/kg, which significantly inhibited histamine-, serotonin-, and bradykinin-induced edema (Figure 4, panels A-F).

Concerning the edema induced by histamine, the EOHc (100 and 300 mg/kg) inhibitory effect extended over the first 60 min (Figure 4A and D). At the peak of histamine-induced edematogenic effect, which occurred at 15 min (Figure 4A), 100 and 300 mg/kg EOHc inhibited 42.88±3.36 and 48.69±2.81%, respectively, of the histamine-induced paw volume increase (103.3±3.606 μL). The cyproheptadine (positive control) inhibited 59.12±6.20% of peak histamine-induced edema (Figure 4A).

In relation to edema induced by serotonin (Figure 4B and E), the inhibitory effect of EOHc (100 and 300 mg/kg) also started in the first 15 min after serotonin administration and was maintained throughout the observation period (120 min). At the edema peak (15 min after serotonin administration), paw volume inhibition corresponded to 22.95±5.17 and 37.25±5.57% (for EOHc 100 and 300 mg/kg, respectively) of the paw volume increase by serotonin (181.7±7.05 µL). The positive control (cyproheptadine) inhibited 74.95±3.16% of peak control edema (Figure 4B).

EOHc (100 and 300 mg/kg) had inhibitory effect on edema induced by bradykinin (Figure 4C and F) observed at 10, 20, and 30 min after injection of bradykinin. At the peak of edema, at the 20th min after bradykinin administration, the inhibition of paw volume corresponded to 57.34±6.54 and 61.1±4.13% (EOHc 100 and 300 mg/kg, respectively) of the increase in paw volume by bradykinin (100.8±5.43 µL).

Comparing the area under the curve for the inhibitory effect of 100 and 300 mg/kg EOHc, the inhibition induced by these doses was, respectively, 34.51±5.19 and 40.37±3.58% for histamine (Figure 4D), 34.42±6.69 and 44.49±4.76% for serotonin (Figure 4E), and 43.66±12.74 and 52.51±1.87% for bradykinin effect (Figure 4F).

### Effect of EOHc on MPO activity

**Figure 5:**
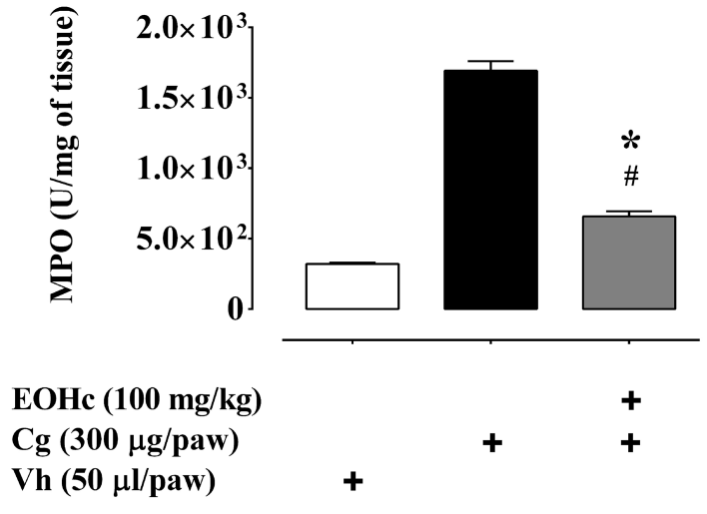
Effect of essential oil of *Hyptis crenata* (EOHc, 100 mg/kg, orally) on myeloperoxidase (MPO) levels. The MPO dosage was made in homogenized paw tissue after 3 hours of edema induced by intraplantar injection of carrageenan (Cg, 300 µg/paw) or Vehicle (Vh, 0.9% NaCl solution, 50 μL/paw) and normalized by mg of tissue. Data are reported as means±SE (n=6). *P<0.05 *vs* control, ^#^P<0.05 *vs* vehicle (one-way ANOVA followed by Bonferroni's test).

As expected, at the inflammatory peak, 180 min after edema induction, the paw edema induced by carrageenan was associated with a significant increase in MPO activity, a marker of neutrophilic infiltration, from 3.21×10^2^±0.0632×10^2^ to 16.93×10^2^±0.5216×10^2^ (U/mg of tissue) (Figure 5). In the group with edema induced by carrageenan pre-treated with EOHc (100 mg/kg) by the oral route, the carrageenan-induced increase of MPO activity was significantly decreased (P<0.05) to about 40% of carrageenan control-induced MPO activity. Although undergoing a significant decline, MPO activity did not return to control levels, observed before carrageenan administration (P<0.05, Figure 5).

## Discussion

The major discovery of this investigation was that EOHc had an antiedematogenic effect in experimental models of acute edema. This EOHc effect was obtained with a great pharmacological efficacy and a conspicuously long duration. Since this is an accepted model of anti-inflammatory activity, the data suggested that EOHc acted through an anti-inflammatory effect. This also showed, for the first time, that the effect of EOHc was very potent to prevent the formation of edema, but also has great efficacy in reversing the already installed edema.

Some EO of aromatic plants have antiedematogenic effects, such as *C. zehntneri* EO (21), *Lavanda augustifolia* EO (22), and *Ocimum basilicum* EO (23). However, it is worth mentioning that for EOHc, the antiedematogenic effect occurs with very small doses (10 mg/kg), representing <0.5% of the LD_50_ (LD_50_ >2.0 g/kg, orally (9)). Moreover, efficacy with such a small percentage of the LD_50_ was not observed for several other EOs, such as *Lavandula augustifolia* Mill EO (21) and *Ocimum basilicum* EO (22).

Previous studies with three species of the genus *Hyptis*, including *H. crenata* show that these plants have common constituents as alpha-pinene, 1,8-cineol, and beta-caryophyllene and point out that these species have important biological activities such as antioxidant and antimicrobial activity (24), in addition to low toxicity (10,24). There are some works showing that monoterpene compounds present in EOHc have anti-inflammatory activity (25–27). The main components of EOHc are monoterpenoid and sesquiterpene compounds (28), such as camphor, alpha-pinene, 1.8-cineole, and beta-caryophyllene (4,10,11,14). Therefore, EOHc, used traditionally for treatment of inflammatory diseases, also has anti-inflammatory activity, as demonstrated here.

The induction of edema by phlogistic agents, such as dextran or carrageenan, is a classical model that allows evaluating substances with anti-inflammatory action. The EOHc showed an antiedematogenic effect in both models, which cause edema by distinct mechanisms. Dextran and carrageenan promote an increase in vascular permeability by different mechanisms. Therefore, analyzing this set of results, it is possible to suggest that EOHc possesses activity on vascular events of inflammation, possibly by inhibition of histamine and serotonin release by mast cells or by neutrophils, and/or by inhibition of action of these substances on receptors (29).

In the carrageenan-induced edema, it was evaluated if the time of administration of EOHc would influence the effect of the oil. Although it inhibited the edema at all times of administration, it was pharmacologically more potent when administered 60 min before induction (Figure 3C and D).

EOHc was more potent in inhibiting the carrageenan-induced edema than that induced by dextran. Oral pretreatment with 30, 100, or 300 mg/kg EOHc inhibited 60, 75, and 80% of carrageenan-induced and 40, 50, and 60% of the dextran-induced peak of edema, respectively. Dextran, a polysaccharide of high molecular weight, induces osmotic and acellular edema, mainly mediated by histamine and serotonin (5-hydroxytryptamine), consequent to the degranulation of mast cells residing in the endothelium of the microvessels (30,31). The histamine and serotonin released, acting on their respective receptors (H1, H2, and 5HT2) (31,32), lead to increased vascular permeability and fluid extravasation. On the other hand, carrageenan, a sulfated polysaccharide extracted from algae, induces an inflammatory response with different phases, with infiltrate containing large numbers of neutrophils and proteins (33–36). In the first phase, which occurs in the first 60 min after the injection of carrageenan, release of histamine and serotonin occurs. In the second phase (61-120 min), release of kinins predominantly occurs, such as bradykinin, and in the third phase (121-180 min) the release of mainly prostaglandin occurs (33–35). EOHc inhibited the edema of cellular nature induced by carrageenan throughout the full time-course.

Since the inflammation induced by dextran involves the release of inflammatory mediators histamine and serotonin and carrageenan also involves bradykinins, the anti-inflammatory effect of EOHc implies at least a partial antagonistic effect of this EO to the edematogenic effect of these inflammatory mediators. This partial antagonistic effect of EOHc to the edematogenic activity of histamine, serotonin, and bradykinin was demonstrated here (Figure 4). Additionally, MPO increase, a parameter related to the inflammatory activity, was partially prevented by EOHc (Figure 5). EOHc inhibitory activity on the edema and MPO activity increase elicited by edematogenic stimuli demonstrated the true anti-inflammatory mechanism of this EO. Although the blockade by EOHc of the edema induced by histamine and serotonin was partial, at 100 and 300 mg/kg, EOHc effect was similar to those of cyproheptadine and indomethacin (Figure 4), demonstrating the efficacy of this EO.

Additionally, carrageenan-induced paw edema lasts for 72 h and after that time only a hypernociceptive process remains (37). Although it was not the object of our investigation to evaluate the entire time course of carrageenan-induced paw edema, we observed that a single dose of EOHc (100 mg/kg) was able to prevent edema increase up to 24 h of observation.

It was not the purpose of this work to fully investigate the mechanism of action for the anti-inflammatory effect of EOHc. However, based on the effects promoted by EOHc - i) inhibition of dextran-induced edema; ii) inhibition throughout the time course of carrageenan-induced edema; iii) inhibition of edema promoted by the inflammatory mediators histamine, serotonin, and bradykinin; and iv) inhibition of MPO activity - it is very likely that the antiedematogenic effect of EOHc was caused by anti-inflammatory effect. The mechanism of this anti-inflammatory effect is, in part, likely to include the inhibition of increased vascular permeability, which occurs upon release of inflammatory mediators, like histamine, serotonin, cytokines, etc.

Other EOs, like *Pterodon Polygalaeflorus* (15), inhibit dextran-induced edema (77.5%) and the first phase of carrageenan-induced edema (76.98%). The authors suggested that this oil might be inhibiting the synthesis, release, and/or effects of histamine and serotonin (15).

In conclusion, we demonstrated that the EO of *Hyptis crenata*, a plant widely used in folk medicine, had anti-edematogenic activity at very low doses compared to its LD_50_, and was likely to be of low toxicity. This EOHc effect, which is interpretable as anti-inflammatory activity due to its inhibitory effects on autacoids and on MPO activity, is consistent with its anti-inflammatory use in folk medicine. The oil showed a potent effect in both edema prevention and reversal of the edema already installed. EOHc efficacy at doses likely to be of low toxicity in humans suggested that this oil has potential for therapeutic use and also that the popular medicinal use of the plant could have a scientific foundation.
